# Editorial: Tissue Resident Memory T Cells

**DOI:** 10.3389/fimmu.2019.01018

**Published:** 2019-05-27

**Authors:** Fathia Mami-Chouaib, Eric Tartour

**Affiliations:** ^1^INSERM UMR 1186, Integrative Tumor Immunology and Genetic Oncology, Gustave Roussy, EPHE, PSL, Fac. de Médecine – Univ. Paris-Sud, Université Paris-Saclay, Villejuif, France; ^2^INSERM U970, PARCC (Paris Centre de Recherche Cardiovasculaire), Université Paris Descartes, Paris, France; ^3^Hôpital Européen Georges Pompidou, Service d'Immunologie Biologique, Paris, France

**Keywords:** TRM cells, antitumor immune response, infectious diseases, T-cell immunity, CD103 integrin, TRM, resident memory T cells

Resident memory T cells (T_RM_) were identified about 10 years ago following the discovery of tissue-resident T cells that do not recirculate. The role of this population of T cells in control of viral infections was rapidly demonstrated. This population is considered to represent a new T-lymphocyte lineage, in that it lacks molecules enabling egress from the tissue and migration to lymph nodes (Klf2, S1Pr1, CCR7, CD62L, etc.) and expresses specific markers of residency (CD103, CD49a, CD69). However, not all T_RM_ cells express these surface markers and their residency feature remains the main characteristic. T_RM_ cells have a distinct differentiation profile dependent on certain cytokines (TGF-β, IL-15, Type I IFN, IL-12) and specific transcription factors (Runx3, Hobit, Blimp-1, Notch, etc.) [Behr et al., (1)]. More than 130 articles were published in 2018 on this population, covering all areas of pathology (infection, allergy, autoimmunity, transplantation, cancer, etc.). The moment thus seemed appropriate for publishing a special issue on this T-cell subset so as to elucidate our current state of knowledge, as well as exploring less frequently addressed issues, such as the specific metabolism of T_RM_ cells (Pan and Kupper), subpopulations of CD4^+^ T_RM_ (Oja et al., Wilk and Mills) and resident lymphocyte populations different from conventional T cells, such as innate lymphocytes or innate-like cells (Chou and Li). The major niches for T_RM_ maintenance and persistence, which is an important issue for this population, are also discussed (Takamura). It is interesting to note that, while this T-cell subset was initially studied in the context of infectious diseases, its role in oncology has recently been demonstrated (2–5). Nevertheless, in the present special issue, the number of articles and reviews dedicated to T_RM_ cells in infection (Wilk and Mills, Morabitoet et al., Muruganandah et al.) is fewer than those dealing with their role in cancer diseases (Oja et al., Blanc et al., Corgnac et al., Dhodapkar, Dumauthioz et al., Smazynski and Webb). This is not surprising; indeed, cancer immunotherapy targets the tumor microenvironment in which T_RM_ cells are located, presumably due to their expression of CD103 integrin, allowing an interaction with tumor epithelial cells expressing E-cadherin (6–11).

The search for cellular targets mediating the therapeutic effects of anti-PD-1 and anti-PD-L1 antibodies is the subject of intense worldwide investigation. This is a medical challenge, and goes hand in hand with the identification of biomarkers predictive of a response to these immunotherapies so as to more effectively select patients likely to respond. The role of T_RM_ has been rapidly addressed; indeed, they represent cells that express high levels of inhibitory receptors (PD-1, Tim-3, etc.) (2, 12), and it has been shown that these lymphocytes proliferate after treatment with anti-PD-1/-PD-L1 (13). Despite expression of high levels of checkpoint receptors, these cells have a cytotoxic capacity, especially after blocking of the PD-1-PD-L1 axis, indicating that they can be reactivated (2, 14). Expression by T_RM_ cells of high levels of granzyme B and TNF-α, as well as the presence of preformed RNA coding for IFNγ, may explain the particular reactivity of these lymphocytes (Behr et al.). A strongly documented hypothesis concerning the mechanism of action of anti-PD-1/-PD-L1 relies on the presence of pre-existing anti-tumor T cells (15, 16). Interestingly, when T_RM_ (CD103^+^CD8^+^ T cells) were separated from the other T cells isolated from the tumor microenvironment, these lymphocytes were enriched in tumor-specific cells (2, 12). In different preclinical tumor models, the presence of these T lymphocytes enables maintaining an equilibrium between the host and tumor, and protects against cancer progression (17). In line with these previous results, mice deficient in T_RM_ cells display accelerated tumor growth (17). In humans, tumor infiltration with this T-cell subset is associated with a favorable prognosis in both univariate and multivariate (2, 12, 14, 18) analyses. T_RM_ cells can be characterized by different techniques (transcriptomic, single cell RNAseq, cytof, etc.) requiring high quality when performing cell isolation. In the present issue, Rissiek et al. report that blocking ARTC2.2 by preventing P2X7 ribosylation improves cell vitality during their *ex vivo* isolation.

Various reviews in this issue are also devoted to a better understanding of mechanisms involved in T_RM_ differentiation *in vivo* and new strategies for inducing them, especially after vaccination (Morabito et al., Muruganandah et al.). T_RM_ cells can be generated from naive T lymphocytes, and a T_RM_ precursor phenotype (KLRG1^low^) has been reported (19). Nevertheless, central memory T (T_CM_) cells and effector T (T_EFF_) cells can also differentiate into T_RM_ cells in peripheral tissue, suggesting a certain plasticity of the pool of memory T lymphocytes (Enamorado et al.). This mode of generation may explain why a common T-cell receptor (TCR) repertoire has been pointed out between T_CM_ cells and T_RM_ cells (20). Differentiation of T_RM_ cells can be inhibited using an anti-TGF-β or an inhibitor of the mTor pathway during T-cell priming (12, 21). Specific parameters might influence generation of T_RM_, such as the high affinity of TCR for the HLA-Class I-peptide complex or a strong inflammatory stimulus (22, 23). In some tissues, but not in others, such as the lung, it has been shown that an inflammatory stimulus without the presence of the antigen may be sufficient to induce differentiation of T_RM_ (5). Finally, in mice, Batf3-dependent type I dendritic cells (DC), corresponding to DNGR-1-expressing DC, appear to be required for priming of T_RM_ (24). In contrast, in humans, CD1c^+^ DC and, to a lesser extent, CD141^+^ DC, play a crucial role in differentiation of T_RM_ cells (25). The need for these local DCs for priming T lymphocytes may explain why the mucosal route of immunization is most effective in priming T_RM_ (26, 27). Vectors targeting certain DC subtypes (4, 28) and some mucosal adjuvants (IL-1β, αGalCer, zymosan. etc.) also boost generation of T_RM_ cells (29–31). The present issue provides the most up-to-date information on T_RM_ cells, but the field is very rapidly evolving. A recent article from Neurath MG's group shows that CD4 T_RM_ cells also play a pathogenic role in models of intestinal inflammation, thus opening up a new field of investigation and indicating a direct role for these lymphocytes in human pathologies (32).

## Author Contributions

All authors listed have made a substantial, direct and intellectual contribution to the work, and approved it for publication.

### Conflict of Interest Statement

The authors declare that the research was conducted in the absence of any commercial or financial relationships that could be construed as a potential conflict of interest.
